# A Ploidy Increase Promotes Sensitivity of Glioma Stem Cells to Aurora Kinases Inhibition

**DOI:** 10.1155/2019/9014045

**Published:** 2019-08-19

**Authors:** Chiara Cilibrasi, Andrèe Guzzi, Riccardo Bazzoni, Gabriele Riva, Massimiliano Cadamuro, Helfrid Hochegger, Angela Bentivegna

**Affiliations:** ^1^School of Medicine and Surgery, University of Milano-Bicocca, 20900 Monza, Italy; ^2^Ph.D. Program in Neuroscience, University of Milano-Bicocca, 20900 Monza, Italy; ^3^NeuroMI, Milan Center of Neuroscience, University of Milano-Bicocca, Dept. of Neurology and Neuroscience, San Gerardo Hospital, 20900 Monza, Italy; ^4^Department of Neurology and Neurosurgery, Montreal Neurological Institute and Hospital, McGill University, Montreal, QC, Canada; ^5^International Center for Digestive Health (ICDH), University of Milano-Bicocca, 20900 Monza, Italy; ^6^Genome Damage and Stability Center, School of Life Sciences, University of Sussex, Falmer, Brighton, UK

## Abstract

Glioma stem cells account for glioblastoma relapse and resistance to conventional therapies, and protein kinases, involved in the regulation of the mitotic machinery (i.e., Aurora kinases), have recently emerged as attractive therapeutic targets. In this study, we investigated the effect of Aurora kinases inhibition in five glioma stem cell lines isolated from glioblastoma patients. As expected, cell lines responded to the loss of Aurora kinases with cytokinesis failure and mitotic exit without cell division. Surprisingly, this resulted in a proliferative arrest in only two of the five cell lines. These sensitive cell lines entered a senescent/autophagic state following aberrant mitotic exit, while the non-sensitive cell lines continued to proliferate. This senescence response did not correlate with TP53 mutation status but only occurred in the cell lines with the highest chromosome content. Repeated rounds of Aurora kinases inhibition caused a gradual increase in chromosome content in the resistant cell lines and eventually caused a similar senescence response and proliferative arrest. Our results suggest that a ploidy threshold is the main determinant of Aurora kinases sensitivity in TP53 mutant glioma stem cells. Thus, ploidy could be used as a biomarker for treating glioma patients with Aurora kinases inhibitors.

## 1. Introduction

Glioblastoma (GBM) is the most common primary malignant brain tumor in adults [1]. Despite multimodality treatments, including surgery, radio- and chemotherapy, outcomes are very poor, with less than 15% of patients alive after two years [2]. A likely cause for recurrence is the presence of a subpopulation of cancer cells with stem-like properties, called glioma stem cells (GSCs) that are resistant to therapies and rapidly repopulate the tumor following the initial treatment [3–5].

GSCs are characterized by their ability to give rise to a differentiated progeny, by their potential to induce glioma-like tumors in mouse xenografts, and by the expression of stem cell markers, such as CD133 and Nestin [6]. A common yet poorly understood feature of GSCs is the elevated chromosomal instability (CIN) [7]. Increases in CIN elicit a p53 dependent response in nontransformed cells [8] but is a common feature of cancer [9].

A variety of mechanisms have been proposed as responsible for CIN, including defects in genes involved in the regulation of the mitotic machinery, such as the Aurora kinases (AurKs) [9]. AurKs are a family of three serine/threonine kinases (AurKs A, B, and C), which play an essential role in controlling mitotic spindle regulation and sister chromatid segregation [10]. AurKs deregulation has been found in a wide range of cancers, including GBM, and is associated with genetic instability and poor prognosis [11–14]. Therefore, they have emerged as attractive therapeutic targets for cancer treatment [15] and several AurKs inhibitors with clinical efficacy in phases I and II of clinical trials have been developed [16].

One of the most clinically advanced compounds is Danusertib (formerly PHA-739358) [17–21], a potent small-molecule 3-aminopyrazole inhibiting the ATP binding site of Aurora kinases. Danusertib has shown considerable therapeutic potential in a wide range of cancers, including advanced solid tumors and leukemias [22–24]. However, to our knowledge, to date there are no reports on the use of Danusertib for the treatment of GBM and its effect on GSCs.

In the present study, we investigated the efficacy of Danusertib on five established GSC lines isolated from GBM patients [7]. The immediate response to Danusertib exposure was uniform among GSC lines and resulted in cytokinesis failure and mitotic exit without division. Surprisingly, only three cell lines responded to this aberrant mitosis by proliferative arrest due to a senescence/autophagy response, while the other cell lines continued to proliferate. Our results suggest that sensitivity to Danusertib in GSCs is determined by a ploidy threshold, beyond which resistant cells enter a p53 independent senescence program.

## 2. Materials and Methods

### 2.1. Cell Lines and Cell Culture Conditions

All the GSC lines (GBM2, G144, G179, G166, GliNS2) were isolated from patients affected by GBM and previously characterized for their stemness properties [25, 26]. GSCs and human foetal neural stem cells (NSCs) (CB660) expansion was carried out as described in [7].

### 2.2. Drug and Treatments

Danusertib (PHA-739358, Selleckem, Houston, Texas, USA) was dissolved in dimethyl sulfoxide (DMSO) to a stock concentration of 10 mM and stored at -80°C. Dilutions to the required concentrations were made using complete medium. Single or two rounds of treatments were performed as reported in Figure 7.

EgV inhibitor STLC (S-Trityl-L-Cysteine, Tocris, Bristol, UK) was dissolved in DMSO at a stock concentration of 50 mM and stored at -20°C. It was used at a final concentration of 5 *μ*M.

DMSO had no effect on the cell survival.

### 2.3. TaqMan Gene Expression Assay

RNA was extracted from GSCs and NSCs using the Direct-zol RNA MiniPrep (Zymo Research, Irvine, California, USA) according to the manufacturer's protocol. RNA quantity and quality were analyzed using a Nanodrop ND-1000 spectrophotometer (Thermo Scientific, Waltham, Massachusetts, USA).

RNA samples were converted into first-strand cDNA using the M-MLV reverse transcriptase (Invitrogen). cDNA was quantified using a Nanodrop ND-1000 spectrophotometer (Thermo Scientific).

TaqMan gene expression assays (Applied Biosystems, Foster City, California, USA) were performed in order to evaluate the expression levels of AurKs A, B, and C in GSCs. GAPDH was used as a housekeeping gene, while CB660 cells were used as normal controls. All the probes used were purchased from Applied Biosystems. Quantitative PCR was carried out using the ABI StepOne (Applied Biosystems), according to the manufacturer's instructions. The cycle conditions were as follows: 2 min 50°C; 10 min 95°C; 40 cycles: 15 s 95°C, 1 min 60°C. Relative gene expression was determined using data from the real-time cycler and the ∆∆C_t_ method. The cut-off values for gene expression fold changes were established at +/- 1,5. The gene expression data were obtained as mean values derived from two independent experiments.

### 2.4. Western Blotting

AurKs protein levels were investigated in cells synchronized by treatment with 5 *μ*M STLC for 24 h and collected by mitotic shake off. Western blotting was performed as already described in [27]. Primary rabbit anti-AurKA (1:500, Abcam, Cambridge, UK) and anti-AurKB (1:500, Abcam) antibodies were used. Mouse anti-*α*-tubulin (1:10000, Abcam) was used as a loading control. Anti-rabbit (1:5000, DAKO by Agilent technologies, Santa Clara, California, USA) or anti-mouse (1:5000, DAKO) HRP-conjugated antibodies were used as secondary antibodies for 1 hour at room temperature. Chemiluminescent detection was performed using an ECL Solution. Emission was captured with radiograph films (Amersham Hyperfilm, GE Healthcare) using an automatic Ecomax X-ray Film Processor.

Beclin and LC3 were detected in GBM2 and G166 cells after one or two rounds of 500 nM Danusertib exposure. Western blotting was performed as already described in [28]. Primary rabbit anti-Beclin (1:1000, Cell signaling technology, Danvers, Massachusetts, USA) and anti-LC3-I and II (1:1000, Cell signaling technology) were used. Mouse anti-GAPDH (1:10000, Santa Cruz Biotechnologies, Dallas, Texas, USA) was used as a loading control. Secondary anti-rabbit (1:2000, Bio-Rad) or anti-mouse (1:2000, Merck, Billerica, Massachusetts, USA) antibodies were used. Proteins were visualized using SuperSignal West Pico or Dura chemiluminescent substrate (Thermo Scientific) with a G-BOX station (Syngene, Bommsandra Bangalore, India).

AurKs, Beclin, and LC3-I and LC3-II bands were quantified with ImageJ (https://imagej.nih.gov/ij/). The experiments were performed at least in triplicate.

### 2.5. MTT Assay

Cell viability was assessed in all the GSC lines by MTT (3-[4,5dimethylthiazol-2-yl]-2,5-diphenyl tetrazolium bromide, Sigma, Saint Louis, Missouri, USA) assay, as already described in [29], after 24, 48, and 72 h exposure to increasing doses of Danusertib (5-50-200-500-1000-5000 nM). MTT was also performed on GBM2 and G166 cell lines after two rounds of treatment with 500 nM Danusertib. The percentage of inhibition was determined as follows: [(treated-cell absorbance/untreated cell absorbance) × 100]. The results reported are the mean values of three different experiments performed at least in triplicate.

### 2.6. Conventional Cytogenetics

Conventional cytogenetics techniques were performed as described in [7] in order to determine the effect of 48 h of treatment with 500 nM Danusertib on the mitotic index and cell ploidy. Briefly, cells were treated with 0,2 *μ*g/ml of Colcemid (Roche) and then harvested and incubated with a hypotonic solution of 0,56% w/v of KCl for 15 minutes at room temperature. Subsequently, cells were fixed with a fixative solution composed of 3:1 methanol:acetic acid. Chromosomes were QFQ banded using quinacrine mustard (Roche) and slides were mounted in McIlvaine buffer. Slides were analysed using Nikon Eclipse 80i fluorescence microscope (Nikon) equipped with a COHU High Performance CCD camera. The mitotic index was evaluated counting the percentage of mitosis scoring at least 1000 nuclei, while ploidy was investigated by evaluating the number of chromosomes/metaphase on 30/50 metaphases. Chromosomes spreads were analysed using the Genikon software. Data were obtained as mean values derived from two independent experiments.

### 2.7. Neurosphere Formation Assay

Cells treated with Danusertib 50 nM and 500 nM for 48 h were retrieved from flasks to obtain a single cell suspension and counted by trypan blue assay. 4x10^3^ cells for each condition were plated in a well of a six-well plate and let to grow for a week. Spheres were counted through the observation at a phase contrast microscopy. Data were obtained as mean values derived from two independent experiments.

### 2.8. Morphological Analysis

Morphological analysis was assessed as already described in [29]. Cells were treated with Danusertib (5-50-200-500-1000 nM) for different times of exposure (24-48-72 h).

### 2.9. Giemsa Staining

Cells grown on coverslips and treated with Danusertib 500 nM for 48 h were fixed with 70% methanol, stained with 10% Giemsa solution (Gibco by Thermo Scientific) in distilled water for 15 minutes, and rinsed off in tap water. Coverslips were let to dry and mounted using Polyvinyl alcohol mounting medium (Merck). Nuclei morphology was evaluated through the observation at phase contrast microscopy. Based on nuclei morphology, cells were divided into four classes: normal nuclei, polymorphic nuclei, multinucleated cells, and micronucleated cells. Representative images were taken for each cell line using 100x oil lens.

### 2.10. DNA Integrity Evaluation

Genomic DNA was extracted from untreated and 48 hs 500 nM Danusertib treated GSCs using the DNeasy Blood & Tissue Kit (Qiagen, Hilden, Germany) according to the manufacturer's protocol.

DNA integrity analysis was performed using an automated tape-based electrophoresis (2200 TapeStation, Agilent technologies) according to the manufacturer's instructions. It automatically calculated the DNA concentration and provided the DNA integrity number (DIN). This numerical assessment of the genomic DNA integrity can range from 1 to 10. DIN>7 indicates highly intact genomic DNA, while DIN<7 indicates a degraded genomic DNA.

### 2.11. Immunofluorescence

Phosphorylated Aurora kinases levels were evaluated after Danusertib 500 nM exposure for 48 h.

Cells were grown on coverslips, synchronized using STLC 5 *μ*M, and fixed with 3.7% paraformaldehyde (Merck) and 70% methanol, for 10 minutes and 1 minute, respectively.

Cells were permeabilized in PBS 0.1% NP40 for 15 minutes, blocked in 3% BSA (Sigma) for 30 minutes, and incubated with mouse anti-*γ*tubulin (1:100, Abcam), rabbit anti-phospho-Aurora A/B/C (1:100, Cell signalling technology), and human anti-Crest (1:100, Antibodies incorporated) primary antibodies for one hour at 37°C. Slides were washed and probed with 488-donkey-anti-mouse (1:2000, Life technologies by Thermo Scientific), 594-goat-anti-rabbit (1:2000, Life technologies by Thermo Scientific), or 670-goat-anti-human (1:2000, Abcam) secondary antibodies for one hour at room temperature. Coverslips were incubated with DAPI for 10 minutes and mounted using the ProLong Diamond mounting solution (Life technologies by Thermo Scientific).

Images were acquired through a 60x oil immersion lens on a Delta Vision Olympus IX70 microscope equipped with a CCD camera using Micromanager software. Images deconvolution was performed using SVI Huygens Professional Deconvolution Software (version 3.5) (Scientific Volume Imaging). For quantitative analysis of phosphorylated Aurora kinases levels, images were imported into Omero software and analyzed with ImageJ software (https://imagej.nih.gov/ij/). Data were obtained as mean values derived from two independent experiments.

### 2.12. Live Cell Imaging

Live cell imaging analysis was performed on GBM2 and G166 cell lines in order to evaluate cell fate when Danusertib 500 nM was added into cell culture.

GSCs were plated in 4 wells chambered slides (Ibidi, Martinsried, Germany) coated with Matrigel and incubated on the Olympus IX71 or IX73 microscopes, equipped with a CCD camera, temperature controller (37°C), and CO_2_ (5%) incubation chamber, soon after the addiction of Danusertib 500 nM.

Images of multiple fields per well were collected every 5 min overnight using a dry 20x objective lens. Images were acquired using the MicroManager software and analyzed by means of ImageJ software.

### 2.13. TP53 Sanger Sequencing

Genomic DNA was extracted from GSCs using the DNeasy Blood & Tissue Kit (Qiagen) according to the manufacturer's protocol. DNA quantity and quality were analyzed using a Nanodrop ND-1000 spectrophotometer (Thermo Scientific).

TP53 exons 5, 6, 7, and 8, representing the mutational hot spot region, were sequenced. The set of primers used to amplify the target DNA and to sequence the DNA was purchased from Life technologies (Supplementary Table S5).

An end-point PCR was performed to amplify the TP53 target loci. DNA quality was examined using an agarose gel electrophoresis. PCR products were purified by means of the EuroSap enzymatic Kit (Euroclone, Pero, Italy). Next, the sequencing reaction was performed using the BigDye terminator v.3.1 kit (Applied Biosystem) according to the manufacturer's protocol. The following thermal cycling conditions were used: 96°C for 40 seconds; 25 cycles composed of 96°C for 10 seconds, 50°C for 5 seconds, and 60°C for 4 minutes; 4°C ∞. The reaction products were purified using EDTA 125 mM pH8 and EtOH. DNA sequencing was performed through a capillary electrophoresis using an ABI-3130 sequencer (Applied Biosystems), with 4 capillaries of 36 cm of length each, loaded with POP-7 polymer. Samples were allowed to run for about 1 h. Data were analysed using the FinchTV software.

### 2.14. Microsatellites Analysis

Loss of heterozygosity (LOH) analysis of chromosome 17 was assessed by means of PCR-based assays, as described in [7]. STR markers used are listed in Supplementary Table S6.

### 2.15. *β* Galactosidase Staining

GBM2 and G166 were grown on coverslips in 6-well plates and treated with 500 nM Danusertib. After one and two rounds of 48 h drug exposure, they were stained using the Cellular Senescence Assay Kit (Cell biolabs, San Diego, California, USA) according to the manufacturer's protocol. Stained cells were mounted using the ProLong Diamond mounting solution (Life technologies).

Random images were acquired on Axio Lb A1 (Zeiss) microscope equipped with an AxioCam ERc 5s (Zeiss, Oberkochen, Germany) and a 40x objective. At least 500 *β* galactosidase positive and negative cells were counted on a computer monitor for each condition and percentages of *β* galactosidase positive cells were calculated. The results are expressed as mean of two independent experiments.

### 2.16. Fluorescence In Situ Hybridization (FISH)

Cells in exponential growth phase were treated with 500 nM Danusertib for one or two rounds of 48 h each. Cell suspension was retrieved from flask and dropped onto glass slides. Cells were fixed with fixative solution composed of 3:1 methanol:acetic acid and FISH analysis on interphase nuclei or metaphase chromosomes was performed using a commercial probe set targeting chromosomes X, Y, 13, 18, and 21 (FAST FISH Prenatal Enumeration Probe kit, CytoCell by Oxford Gene Technology, Begbroke, UK). Signals were counted in at least 50 cells per sample and the number of signals was used in order to predict the cell ploidy. All digital images were captured using a Leitz microscope (Leica DM 5000B) equipped with a CCD camera (Leica Microsystems, Wetzlar, Germany) and analyzed by means of Chromowin software (Tesi Imaging, Milano, Italy). The results are expressed as mean of two independent experiments.

### 2.17. Statistics

Statistical analyses were carried out performing Yates' chi-square test or t-test on raw data, by means of Excel spreadsheet (Microsoft Corporation). p value<0,05 was considered statistically significant.

## 3. Results 

### 3.1. Assessment of Sensitivity and Resistance to Danusertib

**Figure 1:**
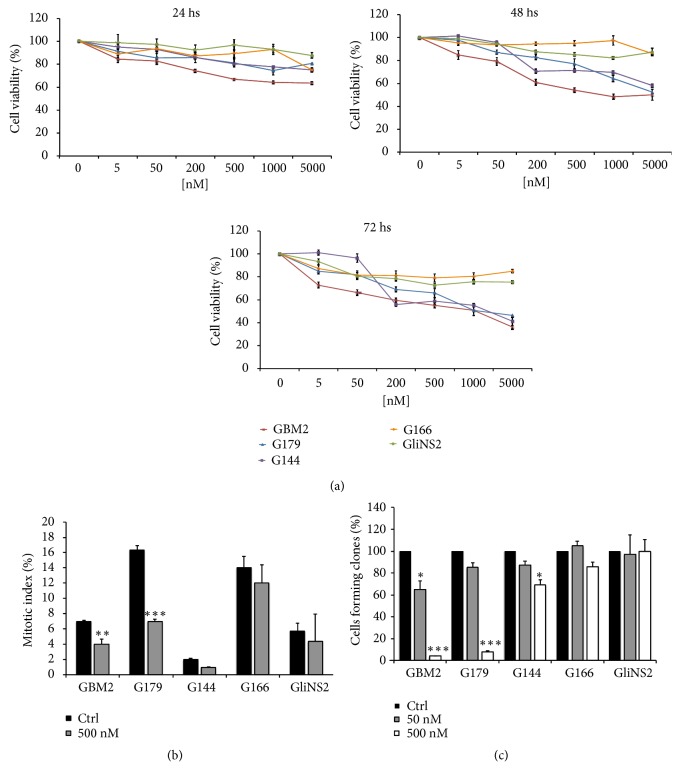
*Different sensitivity of GSCs to Danusertib exposure*. (a) Cell viability was analyzed in five GSC lines by MTT assay after exposure to escalating doses of Danusertib for 24, 48, and 72 h. Results represent the means from three different experiments performed in quadruplicate. Error bars represent SEM. For statistical analysis, see Supplementary Table S1. (b) Cell proliferation was evaluated through the determination of the mitotic index after exposure to Danusertib 500 nM for 48 h. Results are reported as percentages from the means of three independent experiments. Error bars represent SEM. Chi-square test on raw data was performed: *∗∗* p<0,01; *∗∗∗* p<0,001. (c) Clonogenic potential was evaluated after treatment for 48 h with Danusertib 50 nM and 500 nM. Results are reported in a bar graph as percentages of cell forming colonies in the treated samples over matching controls. Results are the means of three independent experiments. Error bars represent SEM. t-test was performed on raw data: *∗* p<0,05; *∗∗∗* p<0,001.

To investigate the potential of Aurora kinases as therapeutic targets in GSCs, we initially verified the transcriptional and protein levels of Aurora kinases in a previously characterized GSCs panel [7]. We found that AURKA and AURKB are upregulated in all the cell lines of the panel and that AurKs are expressed in mitotic GSCs (Supplementary Figures S1A and S1B). Afterwards, we determined the effect of six increasing concentrations of Danusertib on the GSCs viability by MTT assay, after three time points of treatment. Dose-response curves, shown in Figure 1(a), suggest that the Danusertib inhibitory effect was heterogeneous among the cell lines (Figure 1(a), Supplementary Table S1). After 24 h of treatment, we observed a slight decrease of the cell viability in all the GSCs, which was mainly dose-dependent. After 48 and 72 h, this reduction was more evident in GBM2, G179, and G144 cell lines, showing a decrease of around 50% at the highest doses of Danusertib. The reduction values seemed not to be affected by the prolongation of the treatment, remaining almost the same after 48 h and 72 h of exposure. We also assessed the proliferation rates of the cells by mitotic index measurements (Figure 1(b)) and neurospheres formation assays (Figure 1(c)). These data revealed that Danusertib induced a significant dose-dependent reduction of cell viability and self-renewal potential only in some cell lines (Figures 1(b) and 1(c)). Based on the reduction of at least two of the parameters taken into consideration, we classified the cell lines in sensitive (GBM2, G179, and G144) and resistant (G166 and GliNS2).

### 3.2. Danusertib Induces Strong Changes in Cell and Nuclei Morphology in Sensitive GSCs

**Figure 2:**
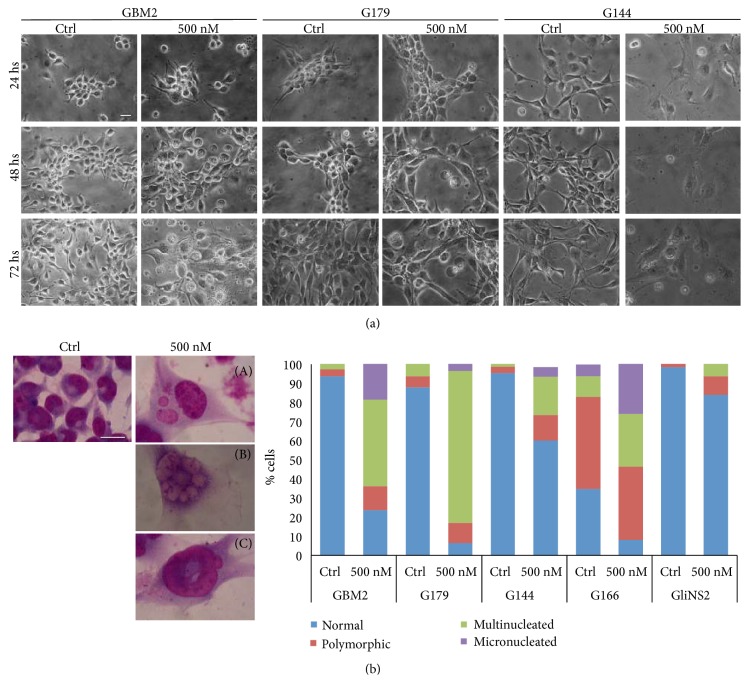
*Danusertib induces evident changes in cell and nuclei morphologies in sensitive GSCs*. (a) Representative images taken by phase contrast microscopy of GBM2, G179, and G144 cell lines treated with 500 nM Danusertib for 24, 48, and 72 h are reported. Drug treatment induced a significant increase in cell size starting from 48 h drug exposure. Scale bar = 100 *μ*m. (b) Representative images of different nuclei shapes detected after 500 nM Danusertib treatment for 48 h and Giemsa staining. Sensitive cell lines highlighted the presence of a huge number of large multinucleated (A) and micronucleated (B) cells and polymorphic nuclei, such as ring-shaped nuclei (C). Scale bar = 100 *μ*m. Quantitative data are reported in the graph. For statistical analysis, see Supplementary Table S2.

In the resistant cell lines, Danusertib did not induce any relevant modification in the cellular shape (Figure S2). On the contrary, Danusertib treatment caused morphological changes in the sensitive cell lines as shown in Figure 2(a). GBM2, G179, and G144 cells showed a dramatic increase in their size starting from 48 h of exposure to Danusertib 500 nM. Hence, we selected 48h and 500 nM, the earliest time point and the lowest concentration at which cells presented evident morphological changes reflecting the Aurora kinase inhibition, for the further experiments.

Additional analysis of nuclei morphology allowed us to distinguish between normally shaped and polymorphic nuclei and multinucleated and micronucleated cells (Figure 2(b), Table S2). A percentage of atypical nuclei, which are a hallmark of cancer, were already present in all the untreated GSCs. In the sensitive cell lines, there was a significant increase in the percentages of polymorphic nuclei and multinucleated cells after the treatment. Moreover, in GBM2, G179, and G144, Danusertib induced also the appearance of micronucleated cells. All these features could mirror the increased cell size previously reported. In the resistant G166, the nuclei morphology was already strongly altered in the control, while in GliNS2 there was only a slight increase in the percentages of polymorphic nuclei and multinucleated cells, but no micronuclei were observed.

### 3.3. Danusertib Inhibits Aurora Kinases and Causes Cytokinesis Failure in Both Sensitive and Resistant Cell Lines

**Figure 3:**
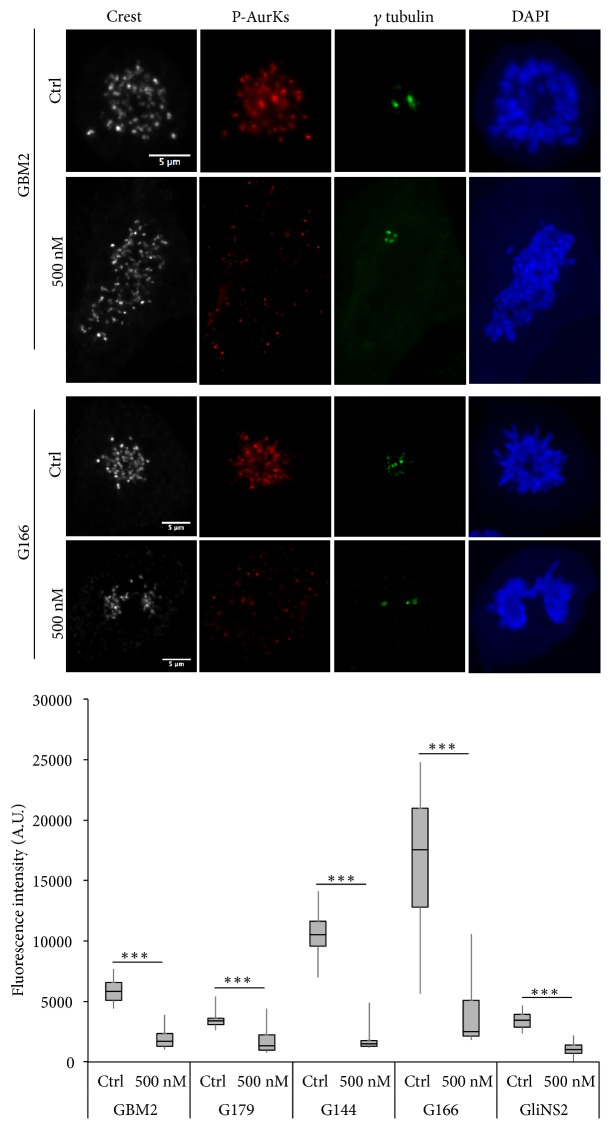
*Danusertib induces a reduction of phosphorylated Aurora kinases in all the GSCs*. Representative images (up) of untreated or 48 h 500 nM Danusertib treated sensitive (GBM2) and resistant (G166) GSC lines, synchronized with STLC and stained for Crest (white), phospho-Aurora kinases (red), *γ* tubulin (green), and DAPI (blue) are reported. Scale bar = 5 *μ*m. Quantification of the pospho-Aurora kinases fluorescent signal in the nuclei of mitotic cells was performed by ImageJ software (bottom). Results are expressed as mean of two independent experiments. At least 25 cells were analyzed in each experiment. Error bars represent SEM. t- test was performed on raw data: *∗∗∗* p<0,001.

A simple explanation for the difference in sensitivity within the GSCs panel could be the different levels of inhibition to AurKs in response to Danusertib, or difference in mitotic aberration following inhibition. In order to ascertain which type of mechanism determines the different behavior, we initially measured these responses to inhibitor treatment by quantitative fluorescence in mitotic cells and by live cell imaging. To assess AurKs activity, we used a phospho-specific antibody that cross-reacts with the T-loop autophosphorylation site in both Aurora-A, B, and C (residues: Thr288, Thr232, Thr198) [30]. We observed a significant reduction of the level of phosphorylated AurKs in all the cell lines suggesting that Danusertib inhibited these kinases in both sensitive and resistant cell lines (Figure 3, Supplementary Figure S3).

**Figure 4:**
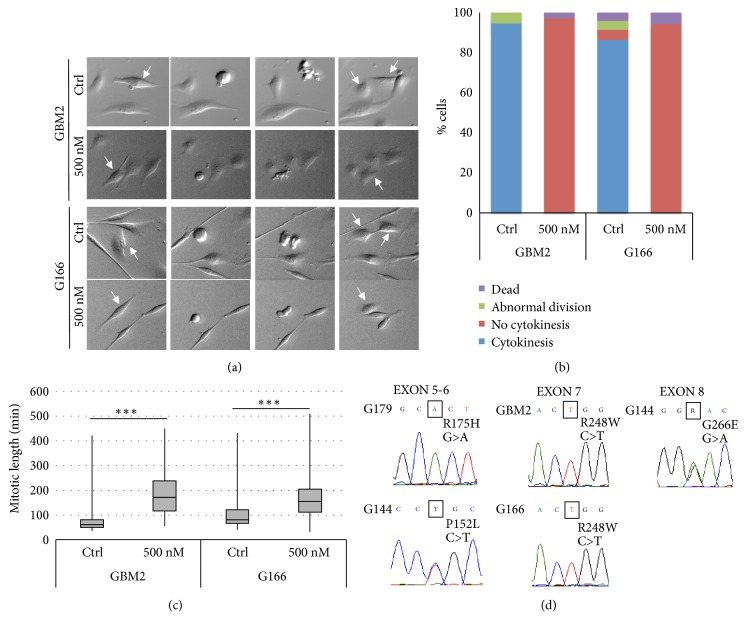
*Danusertib induces a cytokinesis failure and delayed mitotic exit in sensitive and resistant GSCs, independently of the T53 mutational status.* (a) Representative images of dividing cells (indicated by white arrows) detected by means of live cell imaging analysis (see also Supplementary Videos S1, S2, S3, and S4) show that GSCs were able to enter into mitosis after Danusertib exposure but not to divide through cytokinesis. (b) Quantitative data concerning the cells fate after treatment confirm that Danusertib 500 nM inhibited cytokinesis in sensitive and resistant GSCs. The other parameters evaluated, such as the cell death, did not show any relevant variations. Results represent the means from three different experiments. At least 100 cells were analyzed. For statistical analysis, see Supplementary Table S3. (c) Danusertib induced a significant increase in the mitotic length, determined analyzing the live cell movie by means of Image J. Results represent the means from three different experiments. At least 100 cells were analyzed. t-test was performed on raw data: *∗∗∗*p<0,001. (d) Electropherograms of TP53 mutational hot spot regions highlighted that all the GSC lines carried missense mutations in the DNA-binding region of the protein, except for GliNS2 line, which was not affected by any mutation.

In order to better characterize the cell fate induced by Danusertib, we performed live cell imaging analyses in a sensitive (GBM2) and resistant (G166) cell lines (Supplementary Videos S1, S2, S3, and S4). Both cell lines responded to Danusertib with delayed mitotic exit and cytokinesis failure, both hallmarks of Aurora-B inhibition (Figures 4(a), 4(b), and 4(c), Table S3). Thus, the striking difference in cellular survival in response to AurKs inhibition in resistant and sensitive cells is not due to a different level of Auroras inhibition but must be due to a differential response to the failed cell division program in the different cell lines.

### 3.4. Danusertib Induces Ploidy Increase in GSC Lines

Given the highly penetrant cytokinesis defect following Danusertib treatment in both sensitive and resistant cell lines, we expected to observe an increase in ploidy following drug exposure. Measurement of chromosome content identified classes of ploidy and results were grouped according to this classification (Table 1). These data revealed that, even in untreated cells, Danusertib sensitive cell lines have a significantly higher chromosome content compared to the resistant ones. As expected, Danusertib induced an increase in the number of chromosomes in both sensitive and resistant cell lines. Only in GliNS2 cell line, the ploidy remained almost stable. Moreover, this increased DNA content was not fragmented, as demonstrated by the determination of the DNA integrity number (DIN) (Supplementary Figure S4A).

### 3.5. GSCs Response to Danusertib Is Not Dependent on TP53 Mutational Status

An obvious candidate to explain this differential response to polyploidisation is the mutational status of the TP53 gene, which has been shown to trigger cell cycle arrest following increases in ploidy [8]. However, sequencing of the TP53 mutational hot spot regions [31, 32] revealed that both sensitive and resistant cell lines, except for GliNS2, carried missense mutations in the DNA-binding region of the protein (Figure 4(d) and Supplementary Table S4). This strongly suggests that GSCs sensitivity to Auroras inhibition is not TP53 mutational status-dependent. Interestingly, in all cell lines, except for G144, TP53 mutations were in homozygous state, suggesting a loss of heterozygosity (LOH) of whole or part of chromosome 17, as shown in Supplementary Figure S4B.

### 3.6. Danusertib Exposure Induces an Increase in Senescent/Autophagic Cells in Sensitive GSCs

**Figure 5:**
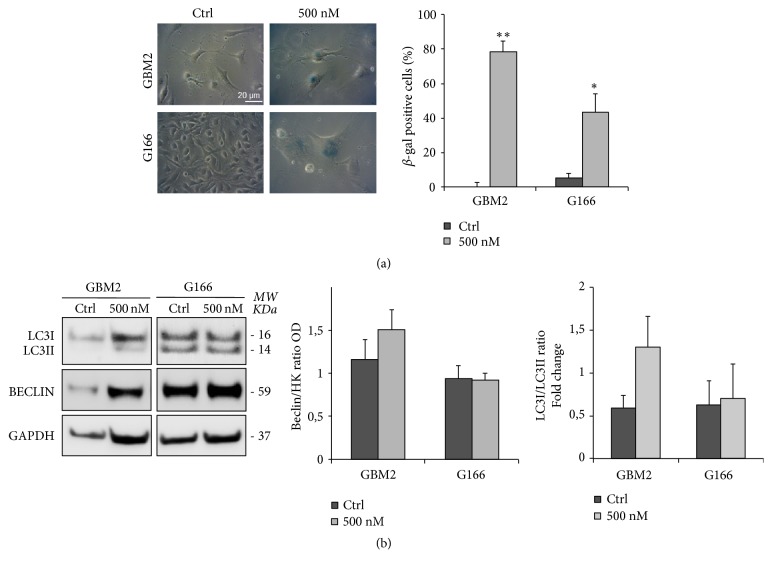
*Danusertib induces senescence/autophagy in sensitive cell line*. (a) GBM2 (sensitive) and G166 (resistant) cell lines were treated with 500 nM Danusertib for 48 h. After the incubation, they were fixed and stained for *β*-galactosidase to evaluate induction of senescence. Representative images showing *β*-galactosidase staining at baseline and after Danusertib treatment are reported. The graph shows the percentages of senescent cells in the two cell lines. Danusertib induced a significant and more relevant increase in the percentage of *β*-gal positive cells only in the sensitive cell line (GBM2). An average of 100 cells/condition/experiment were randomly imaged and scored. Results are representative of three independent experiments. Error bars represent SEM. t-test was performed on raw data: **∗** p<0,05; *∗∗* p<0,01. (b) Beclin level and LC3-I and LC3-II ratio were determined in untreated and 48 h 500 nM treated GBM2 (sensitive) and G166 (resistant) cell lines in order to evaluate the activation of an autophagic process. Representative images of Western blot analysis and quantitative data are reported. Beclin, LCR-I and II levels were determined and normalized on GAPDH. All the values are expressed in Arbitrary Unit (AU). Danusertib induced a non-statistically significant increase of Beclin level and LC3-I and LC3-II ratio only in the sensitive cell line (GBM2). Results are representative of three independent experiments. Error bars represent SEM.

Based on previous studies reporting the ability of Aurora inhibitors to induce senescence [33–35] and the association between senescence and autophagy in polyploid cells [36], we evaluated the levels of *β*-galactosidase and Beclin and the LC3-I/LC3-II ratio in two GSC lines with different sensitivity to Danusertib (GBM2 and G166). Beclin and LC3 have previously been used as markers of autophagy [37]. In particular, Beclin regulates the localization of specific proteins in the early autophagy stages [38], while LC3-I is converted to LC3-II during the activation of this process through lipidation by a ubiquitin-like system, allowing its association with autophagic vesicles [39]. After 48 h of exposure, Danusertib induced a significant increase in the percentage of senescent cells (79%), in the level of Beclin, and in the ratio of LC3-I and II in GBM2. G166 also showed an increase in the *β*-galactosidase positive cells (42%), but there was no variation in Beclin level or in the LC3-I/II ratio (Figures 5(a) and 5(b)).

### 3.7. Cell Ploidy Influences the Different GSCs Response to Danusertib

**Figure 6:**
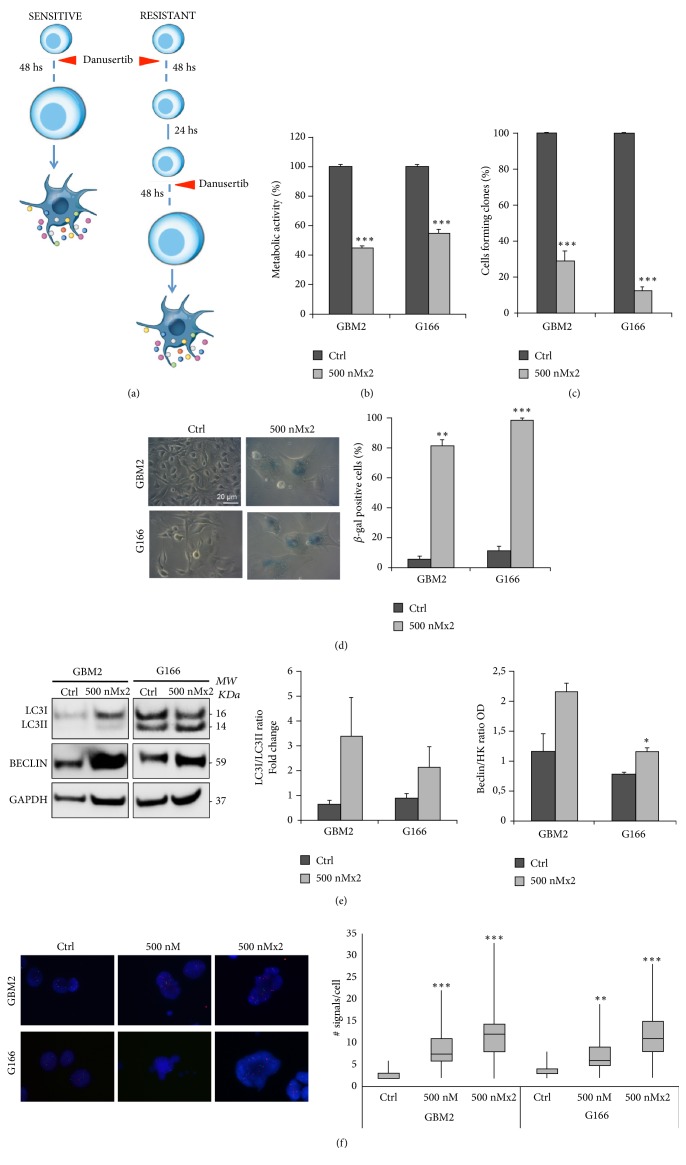
*Multiple rounds of exposure to Danusertib can sensitize the resistant cell line, inducing senescence/autophagy*. (a) Cell lines were subjected to multiple rounds of 48h treatment with Danusertib 500 nM. The sensitization of the resistant cell line (G166) to Aurora inhibition was reached already after two rounds of treatment with Danusertib 500 nM. This was confirmed by MTT assay (b) and the clonogenic assay (c). There was a significant decrease of the cell viability and the clonogenic potential in G166, which was similar to the one observed in GBM2, already after 48 h. Results are the means of three independent experiments. Error bars represent SEM. t- test was performed on raw data: *∗∗∗* p<0,001. (d) GBM2 (sensitive) and G166 (resistant) cell lines were treated with 500 nM Danusertib for two rounds of 48 h each. After the incubation, they were fixed and stained for *β*-galactosidase, to evaluate induction of senescence. Representative images showing *β*-galactosidase staining at baseline and after Danusertib treatment are reported. The graph shows the percentages of senescent cells in the two cell lines. An average of 100 cells/condition/experiment were randomly imaged and scored. After two rounds of exposure to Danusertib, there was a significant increase in the percentages of *β*–gal positive cells also in the resistant cell line (G166). Results are representative of three independent experiments. Error bars represent SEM. t- test was performed on raw data: *∗∗* p<0,01; *∗∗∗* p<0,001. (e) Beclin level and LC3-I and LC3-II ratio were determined in untreated and two rounds treated GBM2 and G166 cell lines in order to evaluate the activation of an autophagic process. Representative images of Western blot analysis and quantitative data are reported. Beclin, LCR-I, and II levels were determined and normalized on GAPDH. All the values are expressed in Arbitrary Unit (AU). After two rounds of Danusertib exposure, an increase of Beclin level and LC3-I and LC3-II ratio was detected also in G166 cell line. Results are representative of three independent experiments. t- test was performed on raw data: *∗* p<0,05. (f) Representative images of FISH analysis performed on GBM2 (sensitive) and G166 (resistant) cell lines after one and two rounds of 48 h treatment with Danusertib 500 nM are reported. Red signals correspond to chromosome 21, while green signals indicate chromosome 13. Scale bar = 100 *μ*m. Quantitative analysis shows that Danusertib exposure induced a steady increase of the number of signals detected in both cell lines. The chromosome content detected in G166 after two rounds of Danusertib exposure is comparable to the one observed in GBM2 cell line already after one round of treatment. Results are expressed as mean of two independent experiments. At least 50 cells were analyzed in each experiment. t- test was performed on raw data: *∗∗∗* p<0,001.

As described above, sensitive and resistant cell lines are characterized by different ploidy, even in the untreated cells. This difference in chromosomes content could be an intrinsic feature of GSCs explaining their distinct fate after Danusertib exposure and suggesting a tolerable threshold in ploidy even in TP53 mutated cells. A direct consequence of this would be that resistant cell lines should also show a sensitization to Danusertib and undergo senescence/autophagy once reaching this threshold. This could be achieved by administering repeated rounds of Aurora inhibition, leading to a steady ploidy increase (Figure 6(a)).

We tested this hypothesis and found that the resistant cell line showed sensitization to Danusertib after the 2nd round of treatment, as demonstrated by the reduction of the cell viability and the self-renewal potential (Figures 6(b) and 6(c)). They underwent a similar senescent and autophagic response, which resulted in a significant increase in the percentage of *β*-galactosidase positive cells (Figure 6(d)), in the Beclin level, and in LC3-I and II ratio (Figure 6(e)). Finally, FISH analyses, performed with a diagnostic kit that detect, among the others, chromosome 21, which is not frequently altered in terms of number of copies in GBM [7, 40], highlighted that, after the 2nd round of Danusertib, the number of chromosomes per cell of the resistant cell line reached the same number observed in the sensitive one, already after 48 h of Danusertib exposure (Figure 6(f)). These data suggest that GBM2 cells reached the ploidy threshold already after 48 h of treatment, while G166 cells only after the 2nd round of Danusertib treatment.

## 4. Discussion

In this study, we analyzed the effects of Danusertib, a pan-Aurora kinases inhibitor with therapeutic potential against a variety of solid cancers [41], on five established GSC lines derived from GBM patients [7]. We discovered a strikingly heterogeneous response among GSC lines, even though Danusertib inhibited Aurora kinases to a similar extent in all the GSCs. Cell viability was significantly reduced only in three cell lines out of five (GBM2, G179, and G144), indicated as sensitive to Danusertib. In these cell lines, Danusertib induced a decrease of the proliferation and clonogenic potential and, above all, evident alterations in the cell and nuclei morphologies, with the appearance of bigger cells and multi- or micronucleated cells with an increased chromosome content. Interestingly, these ploidy alterations were also detected in the resistant cell lines even if they were not linked to relevant morphological alterations, probably because the level of ploidy they reached was not as high as the one detected in the sensitive ones. Live cell imaging analysis shed light on the mechanism underlying the increase in the chromosome number: in both sensitive and resistant cell lines, Danusertib allowed the cells to enter into mitosis but induced a block of the cytokinesis, leading to an increase in ploidy. All these findings confirm previously published data, which demonstrated that Danusertib is able to induce a cell cycle inhibition and endoreduplication in different cancer cells, causing a substantial increase in DNA content with 4N or even >4N cells subpopulations [41].

However, even if a failure of the cytokinesis and an alteration of the cell ploidy was observed in all the GSC lines after 48 h of treatment, sensitive and resistant cells were characterized by a different fate, with only the first ones undergoing a significant activation of a senescent/autophagic process. The senescent phenotype is intriguing and reminiscent of published results [42]. Several studies showed that, after treatment with AurKs inhibition, cancer cells displayed a serial of senescent morphological and functional changes such as enlarged and flattened morphology, increased levels of p21 protein, and enhanced *β*-galactosidase staining, suggesting that, instead of apoptosis, senescence might be the mainly terminal outcome of Aurks inhibition in some tumor types [43]. Interestingly, recent studies also indicated that autophagy, a genetically regulated program responsible for the turnover of cellular proteins and damaged or superfluous organelles, can be a new effector mechanism of senescence, important for the rapid protein remodeling required to make the efficient transition from a proliferative to a senescent state [36]. In our case, the increase in misfolded proteins, caused by a polyploidy-induced proteomic strain, could be at the origin of proteotoxic stress, leading to the activation of an autophagic process, as demonstrated by the increased Beclin level and LC3-I/LC3-II ratio in the sensitive cell line.

A first explanation for cells responding differentially to an AurKs inhibitor has been recently suggested to be depending on the integrity of the p53-p21–dependent postmitotic checkpoint [44], even if several studies yielded conflicting results [45]. The analysis of the TP53 mutational status of our cells showed that both sensitive and resistant cell lines carried missense mutations, supporting the hypothesis that p53 status does not play a key role in determining the differential sensitivity of GSCs to AurKs inhibition.

**Figure 7:**
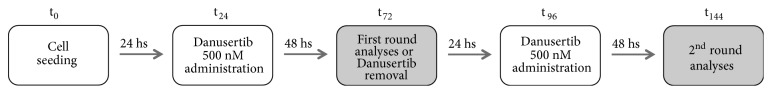
Danusertib administration schedule.

However, an interesting cell feature that emerged to really differentiate between sensitive and resistant cell lines was the chromosome number observed in the untreated cells. We could group the GSC lines by their level of ploidy and correlate this with sensitivity to AurKs inhibition. A straightforward explanation for this result could be the presence of a ploidy threshold, that is intolerable even for p53 negative cell lines. This hypothesis is supported by our experiments with repeated rounds of Danusertib treatment in the resistant cell line (Figure 7). This leads to a further increase in ploidy and ultimately also triggered senescence/autophagy. These results are supported by previously published data, which showed that high amount of chromosome imbalances and alterations are associated with cell growth arrest. Cells with complex karyotype exhibit features of senescence and are even able to produce proinflammatory signals [46].

## 5. Conclusions

Our results suggest that a ploidy threshold is the main determinant of AurKs inhibition sensitivity in TP53 mutant glioma stem cells. Interestingly, previous cytogenetic screenings of GBM specimens have already highlighted that GBMs are characterized by a different chromosomes content [47], supporting the idea that cell ploidy could, indeed, be a promising prognostic factor to predict sensitivity to Danusertib treatment. Further research will be necessary to explore the mechanism of ploidy induced senescence and the precise reason why a particular ploidy threshold appears to trigger this response.

## Figures and Tables

**Table 1:** *Ploidy evaluation after 48 h of treatment with 500 nM Danusertib*. Cells were divided into several classes based on the chromosome content. Danusertib induced an increase in the chromosomes content in both sensitive and resistant cell lines. Italic numbers indicate cell ploidy of untreated cells: sensitive cell lines have a significant higher chromosome content compared to the resistant ones. Chi square test (treated vs untreated): *∗*p<0,05; *∗∗*p<0,01; *∗∗∗*p<0,001. t-test (ctrl sensitive vs ctrl resistant): #p<0,001.

		Class of ploidy (number of chromosomes/metaphase)											
		hypo	hyper	hypo	hyper	hypo	hyper	near	near	near	near	near	near
		diploid	diploid	triploid	triploid	tetraploid	tetraploid	pentaploid	hexaploid	eptaploid	octoploid	nonaploid	decaploid
Cell line	Treatment	35-46	47-57	58-69	70-80	81-92	93-103	104-126	127-149	150-172	173-195	196-218	219-241
GBM2	Ctrl^#^	*0,0*	*0,0*	*0,0*	*10,0*	*0,0*	*0,0*	*16,7*	*73,3*	*0,0*	*0,0*	*0,0*	*0,0*
	500 nM	0,0	0,0	0,0	0,0*∗*	6*∗*	3,0	12,0	36,4*∗∗∗*	39,4*∗∗∗*	0,0	0,0	3,0

G179	Ctrl^#^	*0,0*	*0,0*	*0,0*	*10,0*	*20,0*	*36,7*	*33,3*	*0,0*	*0,0*	*0,0*	*0,0*	*0,0*
	500 nM	0,0	0,0	0,0	3,3	0,0*∗∗∗*	6,7*∗∗∗*	3,3*∗∗∗*	16,7*∗∗∗*	13,3*∗∗∗*	30*∗∗∗*	6,7*∗*	20*∗∗∗*

G144	Ctrl^#^	*0,0*	*0,0*	*2,0*	*23,5*	*56,8*	*15,7*	*2,0*	*0,0*	*0,0*	*0,0*	*0,0*	*0,0*
	500 nM	0,0	0,0	16,7*∗∗∗*	33,3	33,3*∗∗∗*	0*∗∗∗*	0,0	3,3	6,6*∗∗*	3,3	0,0	0,0

G166	Ctrl	*0,0*	*66,7*	*33,3*	*0,0*	*0,0*	*0,0*	*0,0*	*0,0*	*0,0*	*0,0*	*0,0*	*0,0*
	500 nM	0,0	0*∗∗∗*	6,7*∗∗∗*	0,0	0,0	26,7*∗∗∗*	63,3*∗∗∗*	0,0	0,0	3,3*∗*	0,0	0,0

GliNS2	Ctrl	*50,0*	*33,3*	*0,0*	*0,0*	*16,7*	*0,0*	*0,0*	*0,0*	*0,0*	*0,0*	*0,0*	*0,0*
	500 nM	46,7	40,0	0,0	0,0	13,3	0,0	0,0	0,0	0,0	0,0	0,0	0,0

## Data Availability

The data used to support the findings of this study are included within the article.

## Abbreviations

GBM: Glioblastoma
GSCs: Glioma stem cells
CIN: Chromosomal instability
AurKs: Aurora kinases
MTT: 3-(4,5-Dimethylthiazol-2-yl)-2,5-Diphenyltetrazolium Bromide
FISH: Fluorescence in situ hybridization.

## Supplementary Materials

Supplementary Materials
* Supplementary tables*.* Supplementary legends*.* Supplementary Figure S1.* Transcriptional and protein levels of Aurora kinases in GSCs.* Supplementary Figure S2.* Danusertib does not induce evident changes in cell morphology in resistant GSCs.* Supplementary Figure S3.* Danusertib induces a reduction of phosphorylated Aurora kinases in all the GSCs.* Supplementary Figure S4*.* A* Danusertib does not induce any DNA fragmentation in GSC lines.* B* Detailed chromosome 17 LOH mapping of GSC lines.* Supplementary Video S1.* Live cell imaging analysis of untreated GBM2.* Supplementary Video S2.* Live cell imaging analysis of 500 nM Danusertib treated GBM2.* Supplementary Video S3.* Live cell imaging analysis of untreated G166.* Supplementary Video S4.* Live cell imaging analysis of 500 nM Danusertib treated G166.
