# Supplementary material for: A Ploidy Increase Promotes Sensitivity of Glioma Stem Cells to Aurora Kinases Inhibition
Source: J Oncol. 2019 Aug 19;2019:9014045. doi: 10.1155/2019/9014045 (PMC6720056; doi:10.1155/2019/9014045)
Supplement: Supplementary Materials — Supplementary tables. Supplementary legends. Supplementary Figure S1. Transcriptional and protein levels of Aurora kinases in GSCs. Supplementary Figure S2. Danusertib does not induce evident changes in cell morphology in resistant GSCs. Supplementary Figure S3. Danusertib induces a reduction of phosphorylated Aurora kinases in all the GSCs. Supplementary Figure S4. A Danusertib does not induce any DNA fragmentation in GSC lines. B Detailed chromosome 17 LOH mapping of GSC lines. Supplementary Video S1. Live cell imaging analysis of untreated GBM2. Supplementary Video S2. Live cell imaging analysis of 500 nM Danusertib treated GBM2. Supplementary Video S3. Live cell imaging analysis of untreated G166. Supplementary Video S4. Live cell imaging analysis of 500 nM Danusertib treated G166. [file 9014045.f1.zip › 9014045/Supplementary legends.docx]

**A ploidy increase promotes sensitivity of glioma stem cells to Aurora kinases inhibition**

Chiara Cilibrasi, Andrèe Guzzi, Riccardo Bazzoni, Gabriele Riva, Massimiliano Cadamuro, Helfrid Hochegger & Angela Bentivegna

**Supplementary Figure S1. Transcriptional and protein levels of Aurora kinases in GSCs. A.** RT-PCR analysis highlighted that, according to previous published data [1-4], AURKA and AURKB were upregulated in all our GSCs, while AURKC was generally downregulated. Only in G144 cell line, AURKC showed a slight upregulation. Results are expressed as mean of two independent experiments ± SEM. **B.** Western blot analyses were performed on mitotic GSCs, synchronized using STLC. Representative images and quantitative data are reported. AurKs levels were normalized on α-tubulin and all the values were expressed in Arbitrary Unit (AU). Results represent the means from three different experiments. All our GSCs expressed AurkA and AurkB during the mitotic phase. In particular, 4 out of 5 GSC lines showed comparable levels of AurKs. Only GBM2 presented higher AurkA and AurkB protein levels.

**Supplementary Figure S2. Danusertib does not induce evident changes in cell morphology in resistant GSCs.** Representative images taken by phase contrast microscopy of G166 and GliNS2 cell lines treated with 500 nM Danusertib for 24, 48 and 72 hs are reported. Drug treatment did not induce a significant increase in cell size. Scale bar = 100 μm.

**Supplementary Figure S3. Danusertib induces a reduction of phosphorylated Aurora kinases in all the GSCs.** Representative images (up) of untreated or 48 hs 500 nM Danusertib treated GSCs, synchronized with STLC and stained for Crest (white), phospho-Aurora kinases (red), γ tubulin (green) and DAPI (blue) are reported. Scale bar = 5 μm.

**Supplementary Figure S4. A** **Danusertib does not induce any DNA fragmentation in GSC lines.** An automated electrophoretic run of DNA extracted from untreated and 48 h 500 nM Danusertib treated GSC lines was performed and the DNA integrity number was determined. All the DIN values were always >7 indicating DNA integrity. **B Detailed chromosome 17 LOH mapping of GSC lines.** The presence of the same mutations in highly polyploid cells is really unlikely, while is much more likely that malignant cells, which loose the wild-type TP53, have been preferentially selected.

In order to verify the LOH hypothesis and if this alteration was restricted to the TP53 locus (17p13.1) or spread to a bigger region or even to the whole chromosome 17, an LOH analysis was performed using a panel of 4 microsatellite markers mapping on the long or short arm of the chromosome. As expected, G144 cell line showed a retained heterozygosity of the whole chromosome 17. Interestingly G166 cell line presented a whole chromosome LOH as all the microsatellites were homozygous, while GBM2 and G179 cell lines revealed a more restricted LOH. In GBM2 the LOH involved only the distal portion of the p arm of chromosome 17, while in G179 the alteration was probably extended to the whole p arm.

**Supplementary Video S1. Live cell imaging analysis of untreated GBM2.**

**Supplementary Video S2. Live cell imaging analysis of 500 nM Danusertib treated GBM2.**

**Supplementary Video S3. Live cell imaging analysis of untreated G166.**

**Supplementary Video S4. Live cell imaging analysis of 500 nM Danusertib treated G166.**

1. Reichardt W, Jung V, Brunner C, Klein A, Wemmert S, Romeike BF, et al. The putative serine/threonine kinase gene STK15 on chromosome 20q13.2 is amplified in human gliomas. Oncol Rep. 2003;10(5):1275-9.

2. Zhou H, Kuang J, Zhong L, Kuo WL, Gray JW, Sahin A, et al. Tumour amplified kinase STK15/BTAK induces centrosome amplification, aneuploidy and transformation. Nat Genet. 1998;20(2):189-93.

3. Zeng WF, Navaratne K, Prayson RA, Weil RJ. Aurora B expression correlates with aggressive behaviour in glioblastoma multiforme. J Clin Pathol. 2007;60(2):218-21.

4. Klein A, Reichardt W, Jung V, Zang KD, Meese E, Urbschat S. Overexpression and amplification of STK15 in human gliomas. Int J Oncol. 2004;25(6):1789-94.
