# Supplementary material for: A Ploidy Increase Promotes Sensitivity of Glioma Stem Cells to Aurora Kinases Inhibition
Source: J Oncol. 2019 Aug 19;2019:9014045. doi: 10.1155/2019/9014045 (PMC6720056; doi:10.1155/2019/9014045)
Supplement: Supplementary Materials — Supplementary tables. Supplementary legends. Supplementary Figure S1. Transcriptional and protein levels of Aurora kinases in GSCs. Supplementary Figure S2. Danusertib does not induce evident changes in cell morphology in resistant GSCs. Supplementary Figure S3. Danusertib induces a reduction of phosphorylated Aurora kinases in all the GSCs. Supplementary Figure S4. A Danusertib does not induce any DNA fragmentation in GSC lines. B Detailed chromosome 17 LOH mapping of GSC lines. Supplementary Video S1. Live cell imaging analysis of untreated GBM2. Supplementary Video S2. Live cell imaging analysis of 500 nM Danusertib treated GBM2. Supplementary Video S3. Live cell imaging analysis of untreated G166. Supplementary Video S4. Live cell imaging analysis of 500 nM Danusertib treated G166. [file 9014045.f1.zip › 9014045/Supplementary Tables.docx]

| Dose | 5 nM | | | 50 nM | | | 200 nM | | | 500 nM | | | 1000 nM | | | 5000 nM | | |
| --- | --- | --- | --- | --- | --- | --- | --- | --- | --- | --- | --- | --- | --- | --- | --- | --- | --- | --- |
| Time (h) | 24 | 48 | 72 | 24 | 48 | 72 | 24 | 48 | 72 | 24 | 48 | 72 | 24 | 48 | 72 | 24 | 48 | 72 |
| GBM2 | 0,001 | 0,001 | 0,001 | 0,001 | 0,001 | 0,001 | 0,001 | 0,001 | 0,001 | 0,001 | 0,001 | 0,001 | 0,001 | 0,001 | 0,001 | 0,001 | 0,001 | 0,001 |
| G179 | ns | ns | 0,001 | 0,05 | 0,001 | 0,001 | 0,001 | 0,001 | 0,001 | 0,001 | 0,001 | 0,001 | 0,001 | 0,001 | 0,001 | 0,001 | 0,001 | 0,001 |
| G144 | 0,05 | ns | ns | 0,001 | 0,01 | ns | 0,001 | 0,001 | 0,001 | 0,001 | 0,001 | 0,001 | 0,001 | 0,001 | 0,001 | 0,001 | 0,001 | 0,001 |
| G166 | 0,05 | 0,05 | 0,001 | 0,05 | 0,01 | 0,001 | 0,001 | 0,01 | 0,01 | 0,001 | ns | 0,001 | 0,01 | ns | 0,001 | 0,001 | 0,001 | 0,001 |
| GliNS2 | ns | ns | 0,01 | ns | 0,01 | 0,001 | ns | 0,001 | 0,001 | ns | 0,001 | 0,001 | ns | 0,001 | 0,001 | 0,001 | 0,001 | 0,001 |

**A ploidy increase promotes sensitivity of glioma stem cells to Aurora kinases inhibition**

Chiara Cilibrasi, Andrèe Guzzi, Riccardo Bazzoni, Gabriele Riva, Massimiliano Cadamuro, Helfrid Hochegger & Angela Bentivegna

**Supplementary Table S1. Statistical analysis (*p*-values, t-test) of the effects of Danusertib on cell metabolic activity**. p-values are referred to the specific treatment compared to the respective untreated cells. ns = not statistically significant

**Supplementary Table S2. Statistical analysis (*p*-values, t-test) of the effects of Danusertib on nuclei morphology**. p-values are referred to the 500 nM 48 h treatment compared to the respective untreated cells. ns = not statistically significant

|  | **GBM2** | **G179** | **G144** | **G166** | **GliNS2** |
| --- | --- | --- | --- | --- | --- |
| **NORMAL** | 0,001 | 0,001 | 0,001 | 0,001 | 0,001 |
| **POLYMORPHIC** | 0,05 | ns | 0,05 | ns | 0,05 |
| **MULTINUCLEATED** | 0,001 | 0,001 | 0,001 | 0,01 | 0,05 |
| **MICRONUCLEATED** | 0,001 | 0,001 | 0,001 | 0,001 | ns |

**Supplementary Table S3. Statistical analysis (*p*-values, t-test) of the effects of Danusertib on mitotic fate**. p-values are referred to the 500 nM 48 h treatment compared to the respective untreated cells. ns = not statistically significant

|  | **GBM2** | | **G166** |
| --- | --- | --- | --- |
| **Cytokinesis** | | 0,001 | 0,001 |
| **No cytokinesis** | | 0,001 | 0,001 |
| **Abnormal division** | | ns | ns |
| **Dead** | | ns | ns |

**Supplementary Table S4. TP53 alterations found in GSC lines by means of Sanger sequencing.**

|  | **MUTATIONAL HOT SPOTS** | | | |
| --- | --- | --- | --- | --- |
| **Cell line** | **Exon 5** | **Exon 6** | **Exon 7** | **Exon 8** |
| **GBM2** | no alteration | no alteration | **R248W** | no alteration |
|  |  |  | **Variation:** rs121912651 |  |
|  |  |  | **Position** 17:7674221 |  |
|  |  |  | **Alleles** G/A |  |
|  |  |  | **Protein position** 248 |  |
|  |  |  | **Amino acids** R/W |  |
|  |  |  | Missense variant |  |
| **G179** | **R175H** | no alteration | no alteration | no alteration |
|  | **Variation:** TP53_g.12512G>A |  |  |  |
|  | **Position** 17:7675088 |  |  |  |
|  | **Alleles** C/T |  |  |  |
|  | **Protein position** 175 |  |  |  |
|  | **Amino acids** R/H |  |  |  |
|  | Missense variant |  |  |  |
| **G144** | **P152L** |  | **Nucleotide Position: 14181** | **G266E** |
|  | **Variation:** rs587782705 |  | SNP Location: Intron-7 | **Variation:** rs193920774 |
|  | **Position** 17:7675157 |  | SNP Alleles: C/T | **Position** 17:7673823 |
|  | **Alleles** G/A | no alteration | **Nucleotide Position: 14201** | **Alleles** C/T |
|  | **Protein position** 152 |  | SNP Location: Intron-7 | **Protein position** 266 |
|  | **Amino acids** P/L |  | SNP Alleles: T/G | **Amino acids** G/E |
|  | Missense variant |  |  | Missense variant |
|  |  |  | **R248W** |  |
|  |  |  | **Variation:** rs121912651 |  |
|  |  |  | **Position** 17:7674221 |  |
| **G166** | no alteration | no alteration | **Alleles** G/A | no alteration |
|  |  |  | **Protein position** 248 |  |
|  |  |  | **Amino acids** R/W |  |
|  |  |  | Missense variant |  |

| Name | Code | Sequence 5’->3’ |
| --- | --- | --- |
| Exon5-6-FW | Hs00346579_CE | GAGAAAGCCCCCCTACTGCTCA |
| Exon5-6-RV | Hs00346579_CE | CACTTGTGCCCTGACTTTCAACTCT |
| Exon7-FW | Hs00346578_CE | AAAGAGAAGCAAGAGGCAGTAAGG |
| Exon7-RV | Hs00346578_CE | CTTGCCACAGGTCTCCCCAAG |
| Exon8-FW | Hs00346577_CE | TGTTGTTGGGCAGTGCTAGGA |
| Exon8-RV | Hs00346577_CE | CATACTACTACCCATCCACCTCTC |

**Supplementary Table S5. List of TP53 primers**

**Supplementary Table S6. List of STR markers.** Specific information about primers sequences, melting and annealing temperatures can be obtained referring to the UCSC Genome Browser (<http://genome.ucsc.edu/>).

| STR Marker | Position | Sequence 5’->3’ | PCR product size (bp) |
| --- | --- | --- | --- |
| D17S906 | 17p13.1 | Left primer: AGCAAGATTCTGTCAAAAGAG  Right primer: TTCTAGCAGAGTGAAACTGTCT | 335 |
| D17S1159 | 17p13.1 | Left primer: GACAGAAGCACTACACTCAA  Right primer: GTTCCCTGTTTCTGCCTAG | 285 |
| D17S785 | 17q25.1 | Left primer: ATCCCTGGAGAGTGAAAATG  Right primer: AAGGCCAACCTGAAAACTAA | 181-207 |
| D17S787 | 17q22 | Left primer: TGGGCTCAACTATATGAACC  Right primer: TTGATACCTTTTTGAAGGGG | 138-166 |
