# Supplementary figures and images for: A Ploidy Increase Promotes Sensitivity of Glioma Stem Cells to Aurora Kinases Inhibition
Source: J Oncol. 2019 Aug 19;2019:9014045. doi: 10.1155/2019/9014045 (PMC6720056; doi:10.1155/2019/9014045)

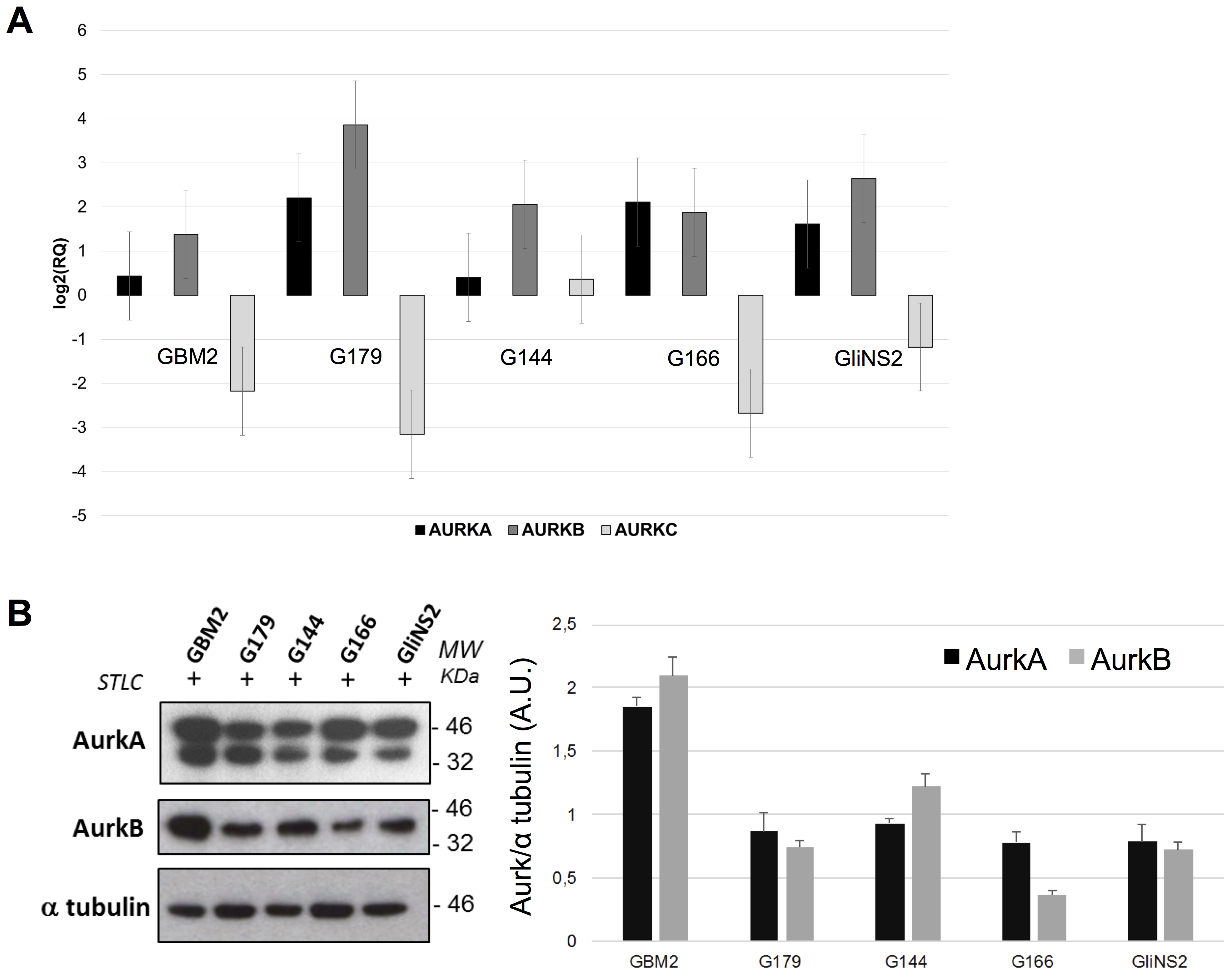

Supplement: Supplementary Materials — Supplementary tables. Supplementary legends. Supplementary Figure S1. Transcriptional and protein levels of Aurora kinases in GSCs. Supplementary Figure S2. Danusertib does not induce evident changes in cell morphology in resistant GSCs. Supplementary Figure S3. Danusertib induces a reduction of phosphorylated Aurora kinases in all the GSCs. Supplementary Figure S4. A Danusertib does not induce any DNA fragmentation in GSC lines. B Detailed chromosome 17 LOH mapping of GSC lines. Supplementary Video S1. Live cell imaging analysis of untreated GBM2. Supplementary Video S2. Live cell imaging analysis of 500 nM Danusertib treated GBM2. Supplementary Video S3. Live cell imaging analysis of untreated G166. Supplementary Video S4. Live cell imaging analysis of 500 nM Danusertib treated G166. [file 9014045.f1.zip › 9014045/Supplementary Figure S1.jpg]

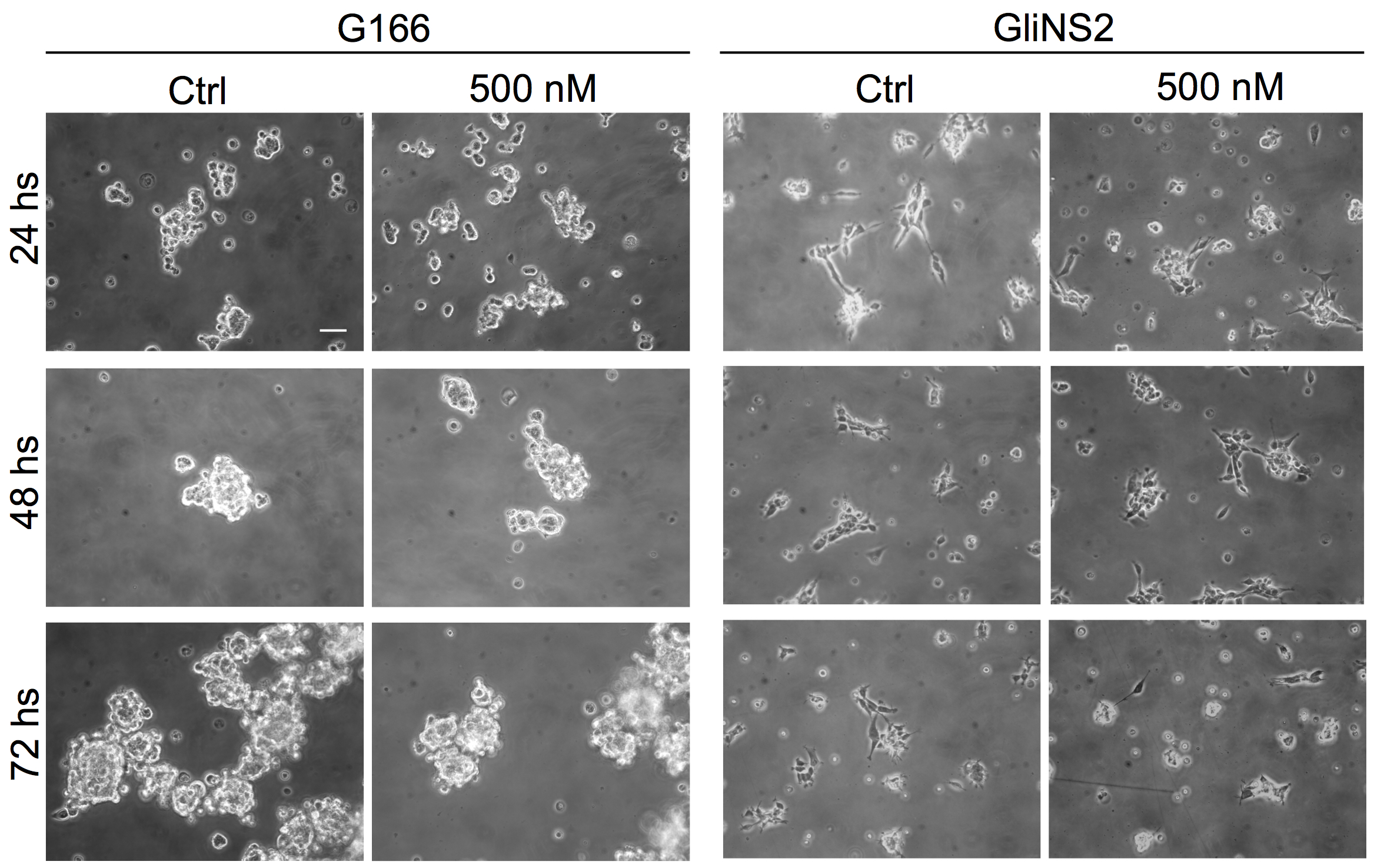

Supplement: Supplementary Materials — Supplementary tables. Supplementary legends. Supplementary Figure S1. Transcriptional and protein levels of Aurora kinases in GSCs. Supplementary Figure S2. Danusertib does not induce evident changes in cell morphology in resistant GSCs. Supplementary Figure S3. Danusertib induces a reduction of phosphorylated Aurora kinases in all the GSCs. Supplementary Figure S4. A Danusertib does not induce any DNA fragmentation in GSC lines. B Detailed chromosome 17 LOH mapping of GSC lines. Supplementary Video S1. Live cell imaging analysis of untreated GBM2. Supplementary Video S2. Live cell imaging analysis of 500 nM Danusertib treated GBM2. Supplementary Video S3. Live cell imaging analysis of untreated G166. Supplementary Video S4. Live cell imaging analysis of 500 nM Danusertib treated G166. [file 9014045.f1.zip › 9014045/Supplementary Figure S2.jpg]

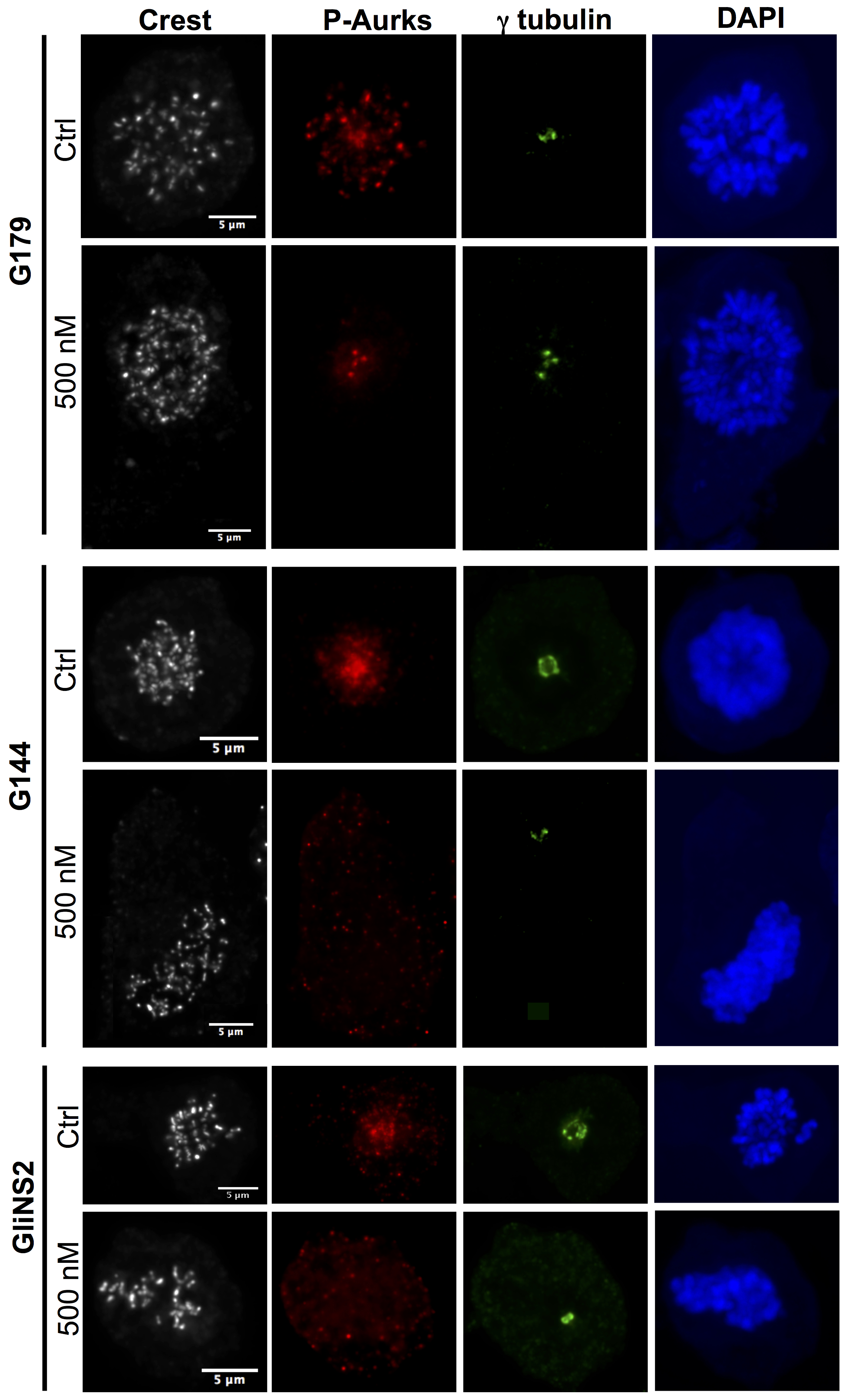

Supplement: Supplementary Materials — Supplementary tables. Supplementary legends. Supplementary Figure S1. Transcriptional and protein levels of Aurora kinases in GSCs. Supplementary Figure S2. Danusertib does not induce evident changes in cell morphology in resistant GSCs. Supplementary Figure S3. Danusertib induces a reduction of phosphorylated Aurora kinases in all the GSCs. Supplementary Figure S4. A Danusertib does not induce any DNA fragmentation in GSC lines. B Detailed chromosome 17 LOH mapping of GSC lines. Supplementary Video S1. Live cell imaging analysis of untreated GBM2. Supplementary Video S2. Live cell imaging analysis of 500 nM Danusertib treated GBM2. Supplementary Video S3. Live cell imaging analysis of untreated G166. Supplementary Video S4. Live cell imaging analysis of 500 nM Danusertib treated G166. [file 9014045.f1.zip › 9014045/Supplementary Figure S3.jpg]

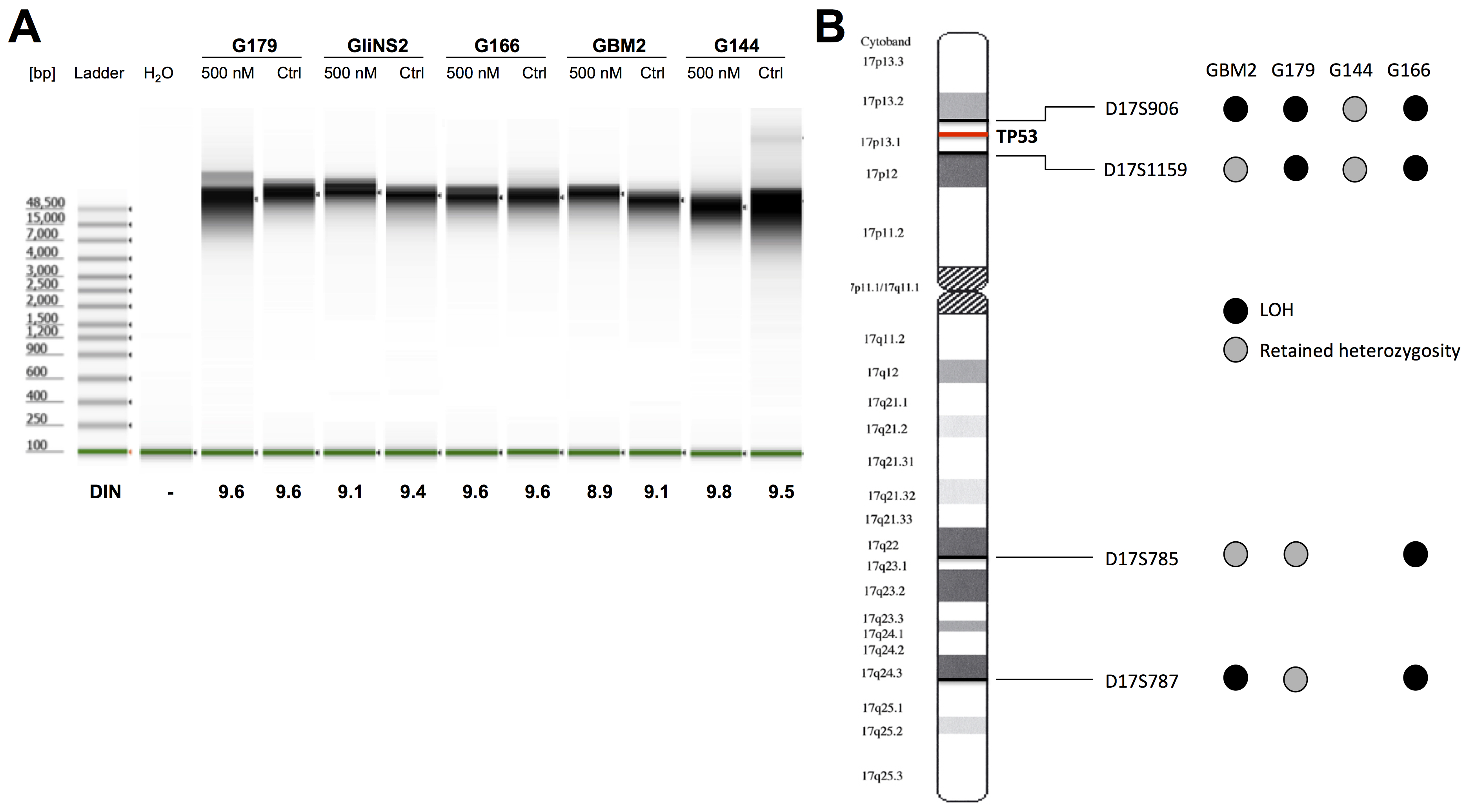

Supplement: Supplementary Materials — Supplementary tables. Supplementary legends. Supplementary Figure S1. Transcriptional and protein levels of Aurora kinases in GSCs. Supplementary Figure S2. Danusertib does not induce evident changes in cell morphology in resistant GSCs. Supplementary Figure S3. Danusertib induces a reduction of phosphorylated Aurora kinases in all the GSCs. Supplementary Figure S4. A Danusertib does not induce any DNA fragmentation in GSC lines. B Detailed chromosome 17 LOH mapping of GSC lines. Supplementary Video S1. Live cell imaging analysis of untreated GBM2. Supplementary Video S2. Live cell imaging analysis of 500 nM Danusertib treated GBM2. Supplementary Video S3. Live cell imaging analysis of untreated G166. Supplementary Video S4. Live cell imaging analysis of 500 nM Danusertib treated G166. [file 9014045.f1.zip › 9014045/Supplementary Figure S4.jpg]
